# Actualizing societal membership in imaginary interaction: The “real construction of society” in the opening of current affairs TV discussion

**DOI:** 10.3389/fsoc.2023.1228498

**Published:** 2023-12-06

**Authors:** Hanna Rautajoki

**Affiliations:** Faculty of Social Sciences, Tampere University, Tampere, Finland

**Keywords:** cultural membership categorization, opening, public sphere, recipient design, societization, structuration, television discussion

## Abstract

The wonder of modern mass-scale society has preoccupied sociological theorists for centuries. How does the whole live on and function? At the extreme of strong empirical traditions, conversation analysis focuses on studying the interactional organization of ongoing action and identities. My article puts these two inquiries together to explore the broader relevancies of situated talk and evidence the skillfulness of social actors in managing multi-scaled cultural memberships simultaneously. Approaching society as a processual accomplishment, this article investigates the instantiation of “societal membership” in a mundane institutional setting of broadcast television. The aim of the article is to experiment with how classical theoretical conceptualization can feed into methodological insight and how detailed empirical scrutiny can enrich our theoretical understanding of the mysteries of modern co-existence. This entails casting an analytic eye on the duality of structure and action. The article re-examines the structural scope of on-site interactional achievements. From an opposite angle, it highlights how integrative societal structures are made real and maintained in the art of interactional encounters. This two-way dynamic is exemplified by scrutinizing a fragment of a televised current affairs program. A set of theoretical key concepts is introduced to shed light on the societal orientations of participants in the opening talk of the program. The opening talk addresses an imaginary audience directly via the camera. It provides a view of the interactional methods used by journalists to invoke relevant identifications for the anticipated recipient at a distance. The encounter is imagined, yet instead of imagining a community in the reception, the analytic focus of the article is on actualizing society in the production of the talk. The spatially and temporally organized societal membership materializes in social relations and interdependencies, which are constituted through intersubjective interpretations, normative positionings, and interactional choices by intentional and knowledgeable actors in the routines of everyday life. The article reverse-engineers the relational framework of the deliberative public sphere enacted in mediated interaction as a collaborative scene of the democratic system. This is achieved by explicating the contextually embedded acts of societization taking place in a journalistically regulated field of participation by means of quasi-interactive public speech.

## Introduction

“The theoretical origins of this enterprise [ethnomethodology] are founded on a basic, indeed classical, sociological question: namely, how can we account for the existence of that thing we call ‘society', defined (in some views) as a systematic, and even functional, organization, which reproduces itself over time? The ethnomethodological ‘take' on this question is that social order can be understood from the point of view of the member of a society, the social ‘actor'.”     (Tolson, 2006, p. 25)

What is “society”, the *membership* of which this passage refers to? How do we access this body of membership empirically? How does such membership materialize in the case of a current affairs discussion program?

Individualization, interdependence, and the major extension of collectives are defining features of modern society (Tönnies, 1887). These circumstances have created a form of social co-existence, the on-site molecular maintenance of which this article explores. In the modern mass-scale condition, imagining unknown others is an integral prerequisite of collective existence, wherein the rise of mass media has served as an important intermediator (Thompson, 1995). Andersson (1991) is known for his ideas about the centrality of imagination at the birth of the modern nation-state, describing how the sense of unity with distant others was facilitated by the spread of literacy and print media. Subsequently, the development of communication media has transformed the spatial and temporal constitution of social life, giving birth to new forms of mediated action and interaction (Thompson, 1995, p. 84–85).

Radio and television engendered a social form of “despatialized simultaneity” and brought about a domain of mediated historicity for people to construct their sense of self, history, and belonging (Thompson, 1995, p. 32–34). The new publicness increased the access and inclusion of the audience yet lacked an opportunity for dialogue. This is not to imply passivity in the uptake, however. Instead, media reception is to be seen as an active, situated, everyday practice coming together as a skilled accomplishment (Thompson, 1995, p. 39–40). Scannell (1989) has also highlighted the “communicative ethos” of broadcasting. It is actively building a communicative relationship with the audience, instilling a sense of familiarity, inclusivity, and sociability in the routines of everyday life (Scannell, 1996; see also Hutchby, 2006). Broadcasting talks to its recipients in conversational ways, inviting their responses and causing individual audience members to assume a group identity in this process (Tolson, 2006, p. 15). Instead of approaching the mass media as a public arena taking place in modern society, this article aligns with the view that it is rather modern society that is taking place and emerging in the communicative practices of this extensive arena (Pietilä, 1999, p. 9). How is one to study this dynamic empirically?

Symbolic forms circulating in the media have the following two cultural characteristics: (1) they are meaningful and (2) they are socially contextualized (Thompson, 1995, p. 10). Either of these angles can be selected to study the relationship between the media and society. The research tradition focusing on the “social construction of reality” is closer to the former. It approaches society as a stock of cultural knowledge that cultivates social roles and world views and thus institutionalizes behavior (Berger and Luckmann, 1967). The research field of social constructionism continued from there and emphasized the relevance of linguistically mediated parallel meaning systems in the constitution and contestation of social realities (Burr, 1995). In this article, I intend to zoom in on the second quality and explore the socially contextualized practices of media communication. In other words, approaching society as a form of action, *societization*, I am interested in the “real construction of society” in broadcast talk (Pietilä, 2011, p. 66). This entails outlining the broader relevancies of talk-in-interaction: to locate, observe, and describe the senses of wider social structure and processes within situated action (Housley and Fitzgerald, 2002, p. 60; see also Lindegaard, 2014). In pursuing the question about the ontology of society, Giddens (1984) called this duality of structure and action *structuration*. In the same vein, the seminal work of Zimmerman and Boden (1991, p. 4) described structures as “something people do”. They state that social structure is not to be seen as something exogenous out there independent of members' activities: it is a practical observable accomplishment of *members of society* (Zimmerman and Boden, 1991, p. 19).

Unraveling the instantiation of society in the media is impossible without acknowledging the relevance of structures. To clarify my analytic angle, the concept of structure can be approached in various ways. In the big picture of sociology, it often refers to social hierarchies, such as the cultural orders around age, race, class, and gender (Zimmerman and Boden, 1991, p. 5). Taking one step further back, it is possible to approach research theories as structures for empirical observations (McHoul, 1994). Even under the umbrella of everyday language use, the word structure has been associated with a variety of things: (1) the organization of talk itself, (2) professional institutions, (3) practical action and reasoning, (4) categorical units, and (5) the just whatness of activities (Psathas, 1995, p. 151–152). My approach in this research comes closest to analyzing how parties in interaction acquire positions as incumbents of broader categorical units. That is to say, I approach cultural structuration from the perspective of *identifications*. In particular, I am interested in the constitution of *societal membership*. “Members of society” are often mentioned in the research literature on social interaction; yet, the body of this membership, society, is seldom subjected to analytic inspection. My article explores the ways in which the current conditions of social co-existence are talked into being in a mundane scene of situated action for participants to engage in.

Live socio-political television discussions represent an enduring program format on Finnish TV. The format dates back to the, 1960s, and over the decades, it has established a lasting position among current affairs programming in the arena of legacy media, most prominently in the supply of the Finnish Public Service Broadcasting Company, YLE. Socio-political television discussions are rooted in the ideals of public service broadcasting, aspiring to support democratic processes and political equality, secure access for all, cultivate cultural diversity, develop domestic culture, and advance enlightenment and education (Hujanen, 2002). I have studied the interactional organization and characteristic features of the program format elsewhere (Rautajoki, 2009, 2012, 2014). My studies have focused on the formation of a “participation framework” (Goffman, 1981; Goodwin, 1987) in a set of programs after the news event of 9/11. I have been mostly fascinated by the role casting of the audience in the programs. The recipient of the talk needs to be imagined in this setting (Goffman, 1981, p. 138). The audience is physically absent yet communicatively co-present. The ways of addressing the audience in media talk ascribe identities to it (Fairclough, 1995, p. 12). Moreover, the normative framework of identifications accomplished in this imaginary encounter is interrelational: the identities of journalists, studio guests, and audience constitute each other in a triangular fashion (Rautajoki, 2009). My research task in this article is to pay attention to the structural implications of these identifications.

I look more closely at the scene of program openings, in which the journalist moderating the discussion speaks to the camera and addresses the audience directly to introduce the topic of the discussion. Opening talk sets the scene for the detailed design of utterances that anticipate and identify the recipients of the program (Sacks et al., 1974), the relevancies of which fall out of scope without the interpretative spotlight of sociological theory. This is to argue that we cannot grasp the full spectrum of multifold memberships enacted and accomplished in the opening lines by confining analytic attention solely to the organization of the on-site interaction and identities. The incentive is to stop and ponder what is, in fact, the gathering imagined and implicated in the design of the talk. To get a better grasp of this relational dynamic, I complement CA and MCA with sociological theories on social action, modern society, and public political discussion. The analytic aim of the article is 2-fold: first, to explicate the detailed organization of address in the opening talk, and second, to deploy theoretical conceptualizations to illustrate the broader relevancies actors orient to and accomplish through their interactional design.

## Theorizing social action on the site of public political arena

Structural theories have dubious connotations in ethnomethodological studies of social interaction. Yet, not all theories are about *a priori* explanations of behavior. Etymologically, derived from the Greek, the word *theori* refers to sight, spectacle, and viewing. A theory is something that enables us to see demonstrable events in social reality in a particular light. In my research, theories are not additional material superimposed on interaction. Instead, I approach theories as spotlights that illuminate participants' observable orientations. They illuminate our view of situated activities. As such, structural theories should not be considered to be alien to CA. Theoretical conceptualizations help us observe cultural recognizabilities. They assist in explicating what exactly is accomplished through the anticipations embedded in the opening address.

**Figure 1:**
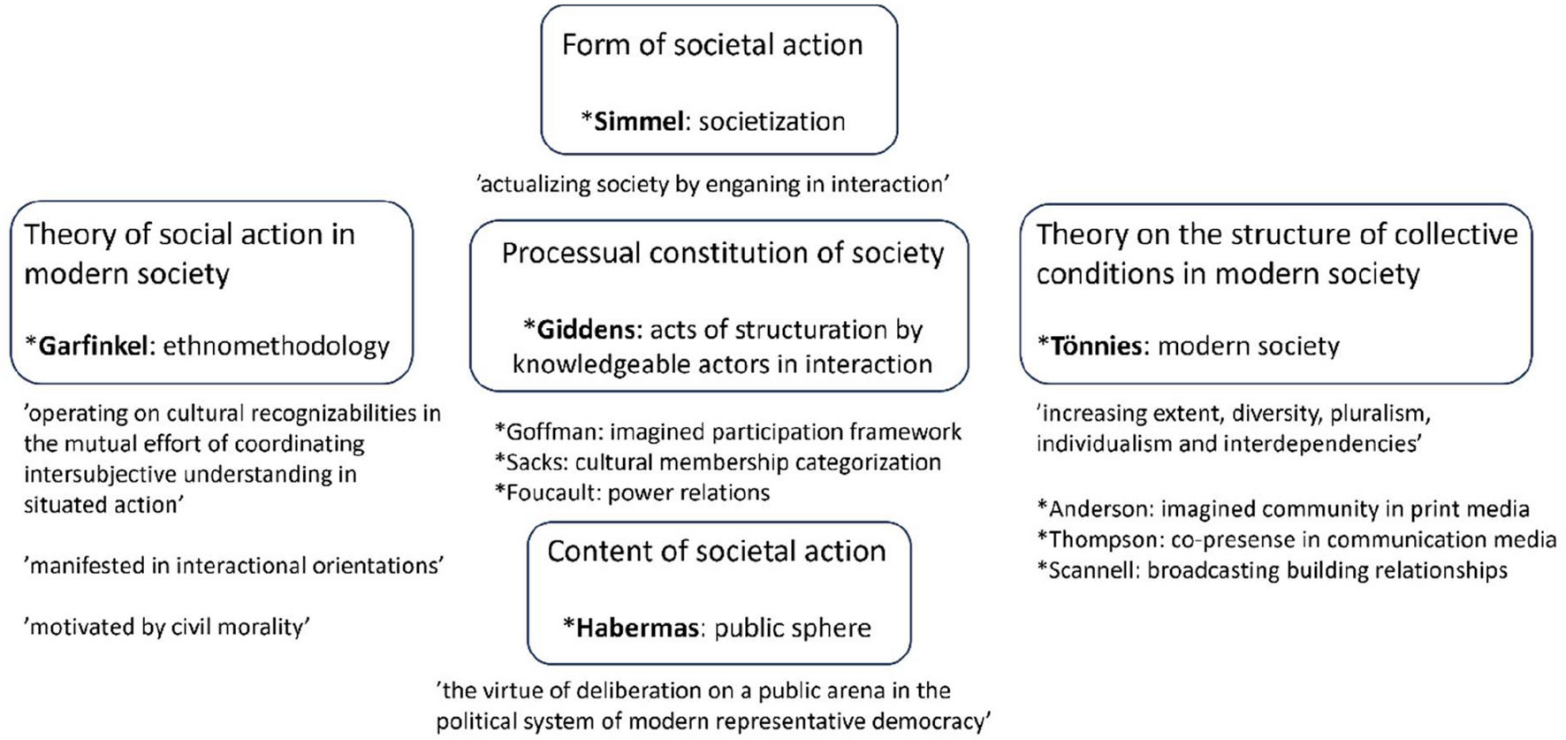
Theoretical cornerstones of the research setting.

The analytic interest of this article lies in the imagined recipients of socio-political discussion programs. Exactly who or what is being addressed in the introductory talk? I aim to delve into the structural premises of societal co-existence and action in modern society. My earlier studies on the program openings aroused my interest in the ontology of society, featuring questions ranging from the lowest common denominator of society to more particular sites of societal action. This article is a theoretical inquiry into concepts to elucidate the scene of concrete intersubjective processes and sites of membership through which the thing we call society emerges and lives on (summarized in Figure 1).

### Societal process—structuration of action

One crucial perspective motivating the need to complement conversation analytic scrutiny with the spotlight of sociological theory is provided by structuration theory (Giddens, 1984). It addresses the question of social ontology through the structural parameters of human action. Structuration theory, interested in the processual constitution of society, aspires to bridge the divide between action and structure in social scientific inquiry and approaches them as a duality. At the core of this duality are spatio-temporal relations (Giddens, 1984, xx–xxi). The structural qualities of any social system only exist if and when the respective forms of social action are chronologically renewed through space and time. This perspective gives primacy to neither the structural determination nor the innate situatedness of action. Instead, structure and action inevitably constitute each other. Accordingly, social institutions evolve in the process of extending particular social activities across broader spans of time and space. A key figure carrying out this “on-site extension” of structural parameters is an active, knowledgeable human actor. The reflexive capabilities of the human actor are an integral part of the recurrent stream of enduring practices pervading everyday life (Giddens, 1984, p. xxii–xxiii). Social actors do not create social practices anew; instead, they continue reinstituting them in the unfolding of action by operating as an actor on that scene. That is, through action, social actors renew the structural conditions that make these activities possible (Giddens, 1984, p. 2).

This idea comes close to the ethnomethodological notion of recognizability (Garfinkel, 1967). The underlying assumption in conversation analysis is that cultural recognizability intermediates intersubjective exchange in social interaction. The empirical focus is on the situated accomplishment of mutual understanding. Yet, to operate on recognizability in social interaction necessitates enduring structures against which an item is recognized and processed as recognizable. Reflexive processing presupposes shared material to be processed. This way, larger frameworks are inevitably present in the situated acts of interaction. The same goes for situated identifications. Structuration theory states that structure is the “virtual order of relationships outside time and place” (Giddens, 1984, p. 304). The stock of cultural recognizability includes actor categories that can be mobilized for identification in situated action. Here again, structures exist only through being actualized by knowledgeable human actors. Actors process and renew enduring identity parameters on-site in particular spatio-temporal locations.

To operationalize the quest into a research setting, the prominence of enduring structures in the constitution of social interaction does not, of course, resolve the dilemma of empirical access in the analysis. The intersective extension of parameters across time and space is difficult to grasp in a situated timeframe. Sacks (1984) concluded in his article “Notes on methodology” that from everything that may have been going on in the interactional setting, the recorded and transcribed talk-in-interaction is something that at least demonstrably took place in the encounter. This does not have to mean disregarding the idea of enduring structures or broader frameworks of action altogether, but it does encourage the analyst to focus on what is available for scrutiny in the intersubjective realm of participants. For the sake of epistemological grounding, the route to exploring the structuration of society in interaction entails tracing observable signs of reflexive knowledgeability surfacing in the orientations of actors. Actualization guides toward relevance here. Any structural parameter, which is to be relevant to the situation at hand, needs to be “procedurally consequential” for the organization of interaction and identifications being actualized on site (Schegloff, 1991). Let us next turn to the question of who exactly is the “we” acting at this actualization in television discussion openings.

### Societal form—practicing societization

Discussion programs are organized around a distributed participation framework, thus involving a distributed constellation of participants (Hutchby, 2006, p. 14). They instantiate a mass-mediated public arena whereby talk is primarily targeted at an imaginary group of individuals. I want to approach the question of an anticipated recipient of the discussion programs from the angle Garfinkel provided in his posthumously published doctoral dissertation (Garfinkel, 2006). He states that a social group, like any social formation, does not consist of persons; it consists of “actors” (Garfinkel, 2006, p. 193). As such, a group should not be approached as an empirical reality. A group does not exist. It is “meant”, that is, made meaningful by the participants to the action. For Garfinkel, a group is “a designator of certain interpretative rules of procedure” (Garfinkel, 2006, p. 199). A functioning group is an aggregate of communicative styles (Garfinkel, 2006, p. 189). Again, its operations are based on cultural recognizability. An effective group occurs with sufficient regularity and summarizes a “designation of social relationship” (Garfinkel, 2006, p. 203). Connecting this to the framework of structuration theory discussed above, to instantiate a group entails enacting the “virtual order of relationships” in ongoing action. To obtain data on group structure, Garfinkel encourages us to “look into the premises of action” (Garfinkel, 2006, p. 198).

To trace the world of premises in interaction, let us next consult ideas evinced by Georg Simmel. Simmel studied the ontology of the phenomenon called “society” (Simmel, 1908). He wanted to extract the defining features of society as a collective constellation, separate from notions like state or nation. Simmel criticized the tendency to treat society as a vessel within which other forms of engagement reside. For Simmel, the broadest idea of society is equivalent to any setting in which people enter into interaction with each other (Simmel, 1999, p. 20–21). All those forms of engagement are what constitutes society: remove them, and no society is left. This perspective treats society as a process that takes place in and becomes real through action. Simmel called this act of engaging with other people “societization”. Furthermore, he differentiated between the form and content of societization. For him, sociology was to concentrate on investigating the pure form of society in the making. Instead of equating society with massive structural entities, he encouraged investigating smaller trivial instances of human relationships and encounters, which flourish endlessly in between large social formations. These microscopic molecular processes of human material represent the actual emergence of society, which is connected and materializes into macroscopic units and formations (Simmel, 1999, p. 37–38).

Diversity and pluralism are core qualities of a human collective in modern society (Tönnies, 1887, p. 29). Unity is not guaranteed by the homogeneity of its components, which means that mutual understanding requires processing. Garfinkel called this continuous effort to work on intersubjective understanding “civil morality”. For Garfinkel, “public civil and secular morality emerges from the collective need to be mutually engaged in practices” (Garfinkel, 2006, p. 9). It is not motivated by anything more than the mutual interest in producing those recognizable orders of practice on which intelligible social life depends (Garfinkel, 2006). This effort at the core of modern societization also underlies the exchanges in television discussions. However, to grasp the specific goals, ideals, and aspirations of the public political debate, one must turn an analytic gaze to the exact *content of societization*.

### Societal content—the premises of a political system as members' accomplishment

For Simmel, the concept of society is both prescriptive and descriptive (Simmel, 1999, p. 27). It means that a group of individuals can function as a society to a lesser or greater extent. The intensity of shared unity and sense of co-determinacy varies. Estimating the degree of awareness and attendance to joint action, the activity framework of current affairs discussion programs appears to implement an enhanced version of societization, in which the intensity of interrelational ties is strongly actualized. The more precise cartography of this intensive coordination is provided by the aims, ideals, and activity roles of the specific situated practice in focus. Reading the orientations of the situation through the lens of structuration theory is to state that, in addition to representing an encounter of institutional interaction in the arena of journalistic practices (here and now in the studio), the talk in television discussion is connected to the structural assumptions about the surrounding political system (out there behind the cameras).

The concept of the public sphere was coined by Habermas (1991) to refer to the historically evolving structural quality of modern representative democracy. The original ideal Habermas describes is based on free and equal individuals gathering at physical locations to hold a critical and rational deliberative discussion on current societal topics, to produce argumentatively achieved, unanimous public opinions, and thus, to intermediate the relationship between the state and civil society. Later research has criticized this conceptualization for its unrealistic, over-idealized, and unequal features (e.g., Calhoun, 1992; Frazer, 1992; Dahlgren, 1995; Thompson, 1995). Habermas (1991) himself was critical of mass media and saw it as deteriorating the public sphere in the direction of institutionally regulated entertainmentization and shallow marketization of democracy focused on vote-catching (Habermas, 1991, p. 163–165). Overlooking the evaluative aspects of this debate, what is more interesting for the purposes of this article is to take distance from the structural idea of the public sphere in the first place and go on to investigate the existence and social constitution of that idea in everyday practices. In theorizing deliberative democracy, Habermas also stated that, even though the pure version of the ideal public sphere may be hard to detect in contemporary society, the institution of public discussion plays a crucial role in bringing about the ideal of *popular sovereignty*, that is, the sufficient inclusion of citizens in the processes of political decision-making and opinion formation, ultimately ensuring the legitimacy of the political system as a whole (Habermas, 1996, p. 299–300).

In his book Modern Social Imaginaries, Taylor (2004) listed *the public sphere* as one of the core ideas to imagine social co-existence in modern society. For Charles Taylor, the concept of modern social imaginary refers to practices and expectations to imagine the interdependencies between separate individuals and the practices to manage that relationship. This specifically concerns a shared understanding of society as a whole, which conditions shared practices and a shared sense of legitimacy (Taylor, 2004, p. 23). The imagination of a broad public following argumentation from a distance and relating it to an extended arena of discussion is one of the particularities of modern society. The peculiarity of that social imaginary is based on the notion of an indefinite space shared by unknown strangers, which covers issues of common concern and yet is set apart from the organs of state politics. The opinion formation taking place in this space serves to regulate, guide, and legitimize political governance (Taylor, 2004, p. 85–87). This imagery of the public sphere has grown to become self-evident to the citizens of today. Yet, in historical view, it has established a new form of collective action and sense of belonging which materializes in radical secular horizontality, detached from religious or other transcendental frameworks, in the worldly time frame, as a result of joint action by principally equal individuals. It is a space that includes all members of society and is also directly accessible to all members without discrimination (Taylor, 2004, p. 157–159).

The public sphere is a good example of a structural entity which does not exist anywhere unless actualized in the “structuration” of situated action. However, it is something that supposedly endures across spatio-temporal locations. I intend to approach this core piece of cultural imaginary suggested in the earlier literature through the lens of scrutinizing “culture in action” (Hester and Eglin, 1997). Again, for the structure to be relevant to the participants, it needs to be procedurally consequential for organizing activities (Schegloff, 1991). Given that the constitution of structural entities is a multi-directional process of emergence, it is important to acknowledge the involvement of simultaneous various orientations. Structural ideas do not reside in spatially nested layers, whereby society provides the largest frame, within which the mass media are located, within which one can find the execution of the idea about a public sphere. Instead, the scaffolding of situated action comprises the relational coordination of parallel cultural assumptions and expectations. To explore the processual formation of structural parameters, it does not suffice to conclude that a mass-mediated program takes place within society. Rather, society, as a process with particular form and content, takes place in the execution of the mass-mediated program and in the unfolding of a concrete interactional encounter (Pietilä, 2011). In this line of thought, my study approaches the society, democratic system, and public sphere as an interactional accomplishment, actualized in particular “discursive spaces, moments and sedimentations” (Housley and Fitzgerald, 2007, p. 189). The object of analysis is then, with the tapestry of cultural imagination in mind, to put it aside for now and trace the markers of cultural sense-making in the concrete details of participants' activities and orientations.

### Research questions

My research task in this article is to analyze the processual structuration of multilayered societal membership. I study the details of interaction in a specific site of societization, namely in the opening lines of Finnish current affairs discussion programs. Viewing the senses of relational unity and mutual co-determinacy in the interactional encounter as an intensifying marker for the societal *form* of co-existence, I relate the *content* of the ongoing societal action (the specific aspirations, aims, and orientations of participants) to the normative cartography of public political discussion embedded in the setting of a journalistically mass mediated program product. The study aims to shed light on the structural relevancies of situated action and identifications by blending the conceptual spotlight of sociological theories with the analytic gaze of conversation analysis and membership categorizations. My study asks:

What kind of momentum in a particular spatio-temporal sphere of action emerges in the formulations of the opening address?
How is the constellation accomplished in the details of interaction?
What kind of identities and expectations are ascribed to the participants in the encounter?

## Materials and methods

The empirical data set of this article consists of three Finnish TV discussion programs broadcast by the Finnish public service broadcasting company, YLE. Briefly, the format of socio-political television discussion is centered around a current topic, which is discussed for between 1 and 2 h from different angles in a live multiparty setting among various studio guests and moderated by one or two journalists. The number of discussants varies from 5 to 23, comprising a combination of experts, politicians, and laypersons. There is no studio audience present. The institutional goal to stage public opinion formation becomes evident in the all-inclusive questions and concluding remarks at the end of the discussion (Rautajoki, 2009). The three current affairs discussion programs all address the same news topic: the terrorist attacks on the United States on 11 September 2001. Four passenger planes were hijacked that day and flown up against buildings, two into the World Trade Center twin towers in New York and one into the Pentagon, the US defense headquarters in Washington DC. One plane crashed, thanks to the actions of the passengers, before reaching the fourth target, the White House in Washington, DC. The terrorist attacks killed close to 3,000 people and shook the world as news of the disaster spread across the globe and the news agencies ended up mediating the scene of the crashing twin towers in real time to people all over the world.

The programs were all broadcast within 3 weeks of the event. In the trajectory of news reporting, they are located in a similar phase: the United States had declared war on terrorism, but there had not yet been any counterstrike on the part of the Americans. The title of the discussion in each program refers to metaphoric war scenarios in the aftermath of the attacks, either the war against terrorism or a religious war between worlds. All the discussions were broadcast live and led by two journalists, one male and one female. The programs all represent the same program format, but the discussions differ in their angle and the combination of guests. For this article, the most interesting aspect is the apparent similarities in the program openings. I will set my analytic eye on the opening lines of the program and view them in the light of the perspectives provided by the sociological theories introduced. The discussions were transcribed and translated into English.

I analyze the data ethnomethodologically, paying attention to the observable details of intersubjective sense-making practices by the participants in social interaction (Garfinkel, 1967). I use methodological tools from both conversation analysis (Sacks et al., 1974) and membership categorization analysis (Sacks, 1972), placing my analytic interest is in the formation of a participation framework, that is, the management and coordination of participant roles in regard to the production as well as the reception of talk (Goffman, 1981; Goodwin, 1987). In mediated interaction, the participation format is distributed and adjusts to the communicative affordances of the medium (Hutchby, 2006, 2014). I am interested in the participatory role of the *audience at a distance*. All talk on TV is primarily targeted at the overhearing audience (Heritage, 1985). However, I will focus on the opening talk of the program, which is specifically addressed to and directed at the audience. I apply the concept of recipient design, incorporating the idea that talk is always designed and structured to target its primary recipient (Sacks et al., 1974). A target of talk who is physically absent must be anticipated, addressed, and invoked in an imaginary encounter. At a distance, the mere involvement of viewers as co-participants in the interaction requires extra effort (Frobenius, 2014). I dig deeper to explore the cultural identities ascribed to the audience in the addresses of the opening talk. A public address, along with its identifications, reaches beyond the interactional organization of activities unfolding in the studio, which accounts for the extended vision provided by the conceptual lenses introduced earlier.

Any opening plays an important role in framing the social encounter at hand (Goffman, 1974, p. 254–255). In television discussions, the opening builds a quasi-interactive relationship with the audience in the form of a monologic speech that unites innumerable people across time and space (Thompson, 1995, p. 84–85). Communicatively, these monologic turns produce “invitations” or first pair parts in the setting of quasi-interactional exchange. Analytically, a monolog lacks the interpretative “next-turn-proof procedure” of the second turn (cf. Sacks et al., 1974), yet the design of these turns can still be analyzed from the perspective of sequential location, organization, and role assignation between parties (Arminen, 2005, p. 118). Interaction in discussion programs is institutional (Drew and Heritage, 1992). It is organized to accomplish specific institutional goals, identities, and inferences (Arminen, 2005, p. 27). However, my primary interest is not in the accomplishment of institutional orders. Instead, I want to highlight the multi-scaled structural relevancies materializing in the orientations of the talk. Just to emphasize, the task is not about discovering a connection between structure and local activities; it is to explicate the methods through which participants manage this structuration. One discursive asset here is the “moral casting” of actors, the skillful regulation of normative frameworks associated with cultural membership categories (Rautajoki, 2012).

I will pursue the identification of the anticipated audience in the programs with a reconsidered model of membership categorization analysis (MCA) (Housley and Fitzgerald, 2002). This is not to fully lose sight of the sequential organization of the action or the emphasis on participants' orientations, the premises guiding CA analysis, but it is to focus analytic attention on the broader relevancies of interactional encounters, following the claim that the wider social structure and its extended processes can also be located, observed, and described within situated action (Housley and Fitzgerald, 2002, p. 60). The pioneer of MCA, Sacks (1972), approached cultural membership categorization as a means for the members of the culture to understand, recognize, and use social actor categories. For him, it was a vital mechanism to produce social orders: he viewed membership categories as cultural “inference-making machines” that are combined with typical features, activities, normative expectations, and interrelations (Sacks, 1995). The interpretative recognizability works both ways here. As a membership category connects to particular “category-bound activities”, an obliging activity orientation can be launched to invoke “activity-bound identifications” (Rautajoki, 2009). Cultural expectations and identities take shape through working on norms in action (Housley and Fitzgerald, 2009; Smith, 2017).

The moral orders and interrelations of categorization provide an important angle for explicating identifications and evaluations in talk (Jayyusi, 1984). However, in my research, I approach the methodical mobilization of normative frameworks slightly differently from Lena Jayyusi, who studied the infusion of description and moral judgment in interpretative activities (Jayyusi, 1984, p. 5–7). Instead of a retrospective evaluative ordering of events and activities, I study the projective use of categorization in forwarded acts of talk-in-interaction intended to address, identify, and engage a recipient (Rautajoki, 2022; Rautajoki and Fitzgerald, 2022). These “normative calls” draw on obliging relationality in the unfolding of action: in the case of this article, involving the audience in the casting of actors, addressing, identifying, and obliging it, and thus coordinating the senses of the social scene.

Another important aspect in managing the participation framework is the prior knowledge of recipients (Goodwin and Goodwin, 1990). The degrees of knowing are one key means of regulating duties, privileges, and hierarchies in interaction (Heritage and Raymond, 2005). These epistemic relations coordinated by participants on site are interconnected with the identities of relevance in the setting (Raymond and Heritage, 2006). The point of departure in my analysis is that the mobilization of cultural membership does not necessitate an appearance of a verbal category; categorical identifications can instead be enacted through action orientations, responsibility calls, and epistemic positionings.

Mass-mediated discourse produces loci of identification for the audience (Housley and Fitzgerald, 2007, p. 198). The conversational qualities of broadcast talk address the co-present audience, eliciting responses from it and suggesting collective identities for it (Tolson, 2006, p. 15–16). The task of empirical analysis is then to ascertain by which means mediated talk relates to its audience inclusively and co-operatively (Hutchby, 2006, p. 11). I will next focus on investigating how the interactional structuration of the broader frameworks of modern co-existence is brought about in the organization of the talk. How do epistemic relations, interrelational membership categorizations and obliging activity orientations turn into “molecular objectives” to explicate a scene of societization?


DATA EXAMPLE 1
TERVO & PÄIVÄRINTA/”TO WAR AGAINST TERRORISM?” 17.9.2001
1     **(1.0) ((J1 in close-up lifts up his gaze from the papers))**
2     **(0.5) ((staring at the camera for a while with a serious face))**
3 J1: **wanted (.) dead or alive, ((said in English originally))**
      wanted (.) dead or alive,
4     **(.) wanted (.) alive or dead, ((the phrase translated into Finnish))**
      (.) halutaan (.) elävänä tai kuolleena,
5     .**hh ↑this is what they used to say (.)**
      .hh ↑näin oli tapana sanoa (.)
6     **in the Wild West of the United States once upon a time,**
      Yhdysvaltain villissä lännessä aikanaan, (.)
7     (.)
8     **this is what the President of the United States says**
      näin sanoo Yhdysvaltain Presidentti
9     **today.**
      tänään.
10    (**0.5) ((intensive indignant look to camera))**


## Acts of societization in the program openings

The data examples below introduce the opening talk of three multiparty TV discussion programs broadcast live by the Finnish public service company, YLE. These excerpts provide brief glimpses of a mundane media setting, which serves well to highlight the degree of multi-scale structuration taking place in a fleeting turn of talk. All the programs deal with the news topic of the 9/11 terrorist attacks. Even though the setup for the three discussions varies in tone, angle, and composition of guests, there are notable similarities transcending the differences in the programs (see also Rautajoki, 2009). I have analyzed the “contextual configuration” (Goodwin, 2000) and the multimodal recipient design of these openings elsewhere (Rautajoki, 2014). For the purposes of this article, I direct my analytic attention to the structural implications of the opening talk with regard to the concepts of structuration, societization, and the public sphere introduced earlier. I will first concentrate on describing what takes place in the opening and then move on to investigate how the organization of talk and interactional choices are indicative of the multilayered structural orientations of the participants? The first of the programs was aired only 6 days after the attacks. J1 and J2 refer to the journalists moderating the discussion.

The journalist (J1) makes a strong gesture to gain the viewer's attention. He raises his eyes dramatically from the papers he is holding in his hand (lines 1–2). Directing the gaze is a common way to point to the addressee of the talk (Goodwin, 1979). For the organization of interaction, gazing has a reciprocal quality. The enacted gaze of the speaker is expected to be returned by the gaze of the recipient to ensure an appropriate state of mutual orientation (Goodwin, 1984). At the start of the talk, the gaze can function as a “summons” to the other party to engage in interaction (Schegloff, 2007). In the program, this serves as a move to establish a quasi-interactional connection with the viewer (see also Frobenius, 2014). The gesture works as the first pair part of the adjacency pair (Schegloff and Sacks, 1973) “gaze–return gaze” and leaves it to the audience to respond accordingly.

The talk itself begins with a dramatic cinematic quotation, first in English and then translated into Finnish (lines 3–4). There is no kind of contextualization for the topic. The assumption is that everybody knows about the news event and recognizes what the journalist is referring to with the quotation, thereby accrediting all viewers with self-evident epistemic competence. Cutting straight to the chase is a powerful way of signaling that this discussion is part of a wider debate extending the boundaries of the immediate interactional encounter. Only one actor category is verbalized in the talk: the President of the United States. The words of the President are delivered in the temporal moment of “now” and equated with the moral anarchy of the Wild West, implying a moral contradiction between that time in history and the proper behavior of a contemporary head of state (lines 6–8). The Wild West is itself an intertextual cultural reference that the recipients are expected to interpret in a similar fashion. The contradiction embedded in the reference sets a worrisome scene with uncertain consequences to be tackled in the program.


DATA EXAMPLE 2
A-TALK/”TO WAR AGAINST TERRORISM” 19.9.2001
1     **((journalists stand behind a table the camera is sliding toward))**
2 J1: .**hhh good (.) Wednesday evening, (0.2). thhh now one can perhaps say that**
      .hhh hyvää (.) keskiviikkoiltaa (0.2) .thhh nyt voi kai sanoa että
3     **(.) the world is holding its breath..hh the United States (.) is preparing**
      (.) hh maailma pidättää hengitystään. hh Yhdysvallat (.) valmistelee 
4     **(.) a counter attack, (.).thh and now people are waiting (.) where (.)**
      (.) vastaiskua, (.).thh ja (.) nyt odotetaan hh minne hh (.) milloin 
5     **when and (.) how..thh (.) but (.) >at the same time<** **(.) there have**
      ja (.) miten..thh (.) mutta (.) >sama an aikaan< (.) maailmalla on 
6     **also (.) been questions raised in the world (0.2) on** **(0.2).thh where the**
      myös (.) noussut kysymyksiä (0.2) sii tä (0.2).thh missä
7     **evidence is against Bin Laden, (.).thh and (.) on (0.2) whether this**
      ovat todisteet Bin Ladenia vastaan, (.).thh ja (.) siitä (0.2) johtaako 
8     **all (.) will lead into (.) a circle of revenge.**
      tämä (.) kaikki (.) koston kierteeseen.
9 J2: .**hh this has also preoccupied Western European <countries> who have promised**
      .hh se askarruttaa myös Länsi-Euroopan <maita> jotka ovat antaneet täyden 
10    **full (.) political support (0.2) for the United States, =>and are now**
      (.) poliittisen tukensa (0.2) Yhdysv alloille, = >ja miettivät nyt<
11    **wondering<.hhh what kind of a war against terror ism they are (.) committing**
      .hhh minkälaiseen sotaan terrorismia h vastaan ne ovat (.) 
12    **themselves to, (0.5).hhh Chirac (.) of France just visited Washington**
      sitoutumassa, (0.5).hhh Ranskan (.) Chirac kävi juuri Washingtonissa 
13    **hhhh Blair of Britain is about to travel there, (0.8) here come (0.2)**
      hhhh Britannian (.) Blair on sinne menossa, (0.8) tässä (0.2) 
14    **our correspondents' (.) reports on, (0.5) what the attitudes look like**
      kirjeenvaihtajiemme (.) raportit siitä, (0.5) minkälaisia 
15    **in the three biggest (.) Western European countries.**
      ovat asenteet kolmessa tärkeimmässä (.) länsieuroopan maassa. 
16    **(1.2) ((journalists looking to camera seriously))**


The choice of a key actor category in the talk, being the President, the leader of the state-level response to the attacks, is no coincidence. It allows other parties to take their positions in relation to this leader, providing the journalist with an opportunity to exercise the journalistic ideal of critically surveilling the power holders. For the audience, the President is presented as a figure who plays the leading role in determining the fate of the Western world. Even though the President represents another nation, the concern is introduced as shared. The facial expression and intense look of J1 to the camera at the end, together with an arresting tone of voice, indicate moral indignation (line 10). The relatively long duration of the look directed straight to the camera makes it appear as another example of the first pair part in which the indignation is provided for the viewer as a gesture to be responded to Peräkylä and Ruusuvuori (2012). The recipient is invited to take a stance on the matter.

The second of the programs was broadcast 8 days after the attacks. It is slightly less confrontational in its introduction to the topic of the day. The two journalists stand still behind a table as the camera slowly approaches them, while one of them begins to speak. The opening starts with a greeting (line 2). This instantly implies that the broadcast is live and the audience is facing the journalist in real time. This empty first pair part, a greeting with no opportunity to respond to it properly, is a familiar practice in news broadcasts. The preference structure (Pomerantz, 1978) of greetings invites the viewer to engage in interaction even if no overt response is feasible (Tolson, 2006, p. 27). A temporally marked greeting is one way of accomplishing a quasi-interactional relationship and connection to the viewer.

The verbal formulation of the opening starts with the word “now” (line 2), emphasizing the acuteness of the topic. The “world holding its breath” signals the dramatic nature of the scene confronting the world, even though the expression is markedly mitigated. The world shares a fate and is waiting in anticipation. The opening sums up the phase of the news situation but provides no contextual information on the news event. It is assumed to be self-evidentially familiar to all and anyone watching the program. Again, the shared epistemic competence assigned to all the participants postulates an extended arena of discussion, of which the program is only a part. It also assumes that viewers share a commitment to get concerned. The situation is characterized as incomplete; there are activities going on even as they speak. Several open questions are listed (lines 5–8). The questions are posed by the “people”, and they have been wondered about “in the world”. The generalized collective origin of the questions paves the way for the viewer to relate to them. The questions communicate reservations regarding possible reprisals. Weak evidence and risk scenarios are played out in pondering about the decision.

The Western European countries are paraded as a “reference group” for the country of Finland to observe and identify with (Hyman, 1942; Pi Ferrer et al., 2019). There is a person reference to the leaders of these countries who are “one the move”. The reference is made rather loosely on a surname basis only, assuming that the audience knows who Chirac of France and Blair of Britain are (lines 12–13). Toward the end, the journalists lead the way to video inserts from “their correspondents” (line 14) to sum up the attitudes in the most important countries. Again, being alert and following the actions of the leaders exercises journalistic ideals. Another layer of appropriate journalistic response is to provide citizens with information from the wider world first hand, through their own correspondents, afforded by YLE as a big news house. However, the phrase “attitudes in the countries” does not contemplate the thoughts of the leaders. The generalized angle of the “country as a whole” can be interpreted as an inclusive invitation for viewers of the country being addressed to engage in deliberation on open questions and uncertainties.


DATA EXAMPLE 3
AJANKOHTAINEN KAKKONEN/”WAR BETWEEN
WORLDS” 2.10.2001
1      **(7.0) ((journalist walk up to the arena discussants are gathered to))**
2  J1: **the whole (.) world is holding its breath and waiting** for the United States'
       koko (.) maailma pidättää henkeään ja odottaa Yhdysvaltain
3      **revenge on the >terrorist< attack three weeks ago, .hhh now people have **
       kostoa kolmen viikon takaiseen >terrori<-iskuun,.hhh nyt on jo
4      **already started wondering, (.) why the counter-strike is delayed. **
       alettu ihmetellä, (.) miksi isku viipyy.
5  J2: **the war against terrorism has already (.) started, there has evolved **
       mt' sota terrorismia vastaan on jo (.) käynnistynyt, Yhdysvaltain
6      **an <unholy> union around the United States where (.) one (.) **
       ympärille on syntynyt <epäpyhä> liitto jossa (.) yhtenä (.)
7      **supporting pillar is the EU and (.) along with it Finland. **
       tukipylväänä on EU ja (.) sen mukana suomi.
8  J1: **when Finland is involved (.) in the battle against terrorism.hh then (.) **
       kun (.) suomi on mukana (.) terrorismin vastaisessa taistelussa.hh niin
9      **what exactly (.) is Finland fighting for. (0.5) is (.) hatred and (.) **
       (.) minkä puolesta (.) suomi taistelee. (0.5) onko (.) viha ja (.)
10     **revenge justified, (.) is a circle of revenge (.) necessary. **
       kosto oikeutettua, (.).h onko ↑koston kierre (.) välttämätön.
11 J2: **is there emerging a new >*****fr*****ontline< Christians against (.) Muslims, **
       onko nyt yy syntymässä uusi >rintama<kristityt vastaan (.) muslimit,
12     .**hhh what says Christianity, (.) what says (.) Islam about killing, **
       .hhh mitä sanoo kristinusko, (.) mitä sanoo (.) islam tappamisesta,
13     .**hh discussing tonight (.) Muslims and Christians, (.) **
       .hh keskustelijoina tänä iltana (.) muslimeja <ja> kristittyjä, (.)
14     **parties of concern and (.) parties with expertise. **
       asian>osaisia<ja (.) asiantuntijoita.
15     **(0.5) ((journalists smiling to camera))**


The third of the programs was broadcast 3 weeks after the terrorist attacks, which accounts for the sense of crisis having slightly subsided in the tone of the opening. The physical set-up of the studio forms an arena of discussants sitting in the studio in concentric circles, into the middle of which the journalists walk to deliver their opening words. Before the entrance walk, the camera angle leads the viewer into the studio by sliding over the circle of discussants from the corner of the backstage furniture as if walking into the studio by the same route as the studio guests took some minutes before. The word focalization is used in narratology to study the perspective of narrative (Genette, 1979). Its further elaboration has included the aspects of vision and perception in focalization, considering it as an angle of perception that postulates the point of origin, the one who sees (Jahn, 1996). The circuitous camera angle running through the backstage sets the viewer alongside the other discussants waiting in the studio. The two journalists walk-in only after the viewer has arrived in the arena. The entrance is not as interactive as in the first two programs. However, it appears to pursue active involvement and inclusion of the audience.

The talk directed straight at the camera repeats the phrase “the world holding its breath and waiting in anticipation” upfront (line 2). There is no contextualization for the news event except for the intervening period of 3 weeks. Everybody is supposed to know the background. Again, there is the generalized collective “people” (lines 3–4) wondering about the progress of ongoing events, which makes it easier for the audience to join in and identify with. This is further strengthened by casting Finland in the role of a moral actor on the scene of uncertain and open events (lines 8–10). Finland is referred to as a metonymic whole, and again, open questions are used rhetorically and inclusively to activate alertness in the viewers facing alternative pathways. The talk presents the appropriateness of Finland to be on the line, which enhances the collective pressure to take a stance. It concludes by speculating about a confrontation between Muslims and Christians, which makes an intertextual cultural reference to the famous work by Samuel Huntington on the clash of civilizations. The reference is dramatized and leans on familiarity. It is referred to as an angle everybody knows without further explanation. The institutional principle guiding this set-up for the discussion resonates with the journalistic ideal to facilitate open debate and dialogue: a mixed group of people, both the aforementioned parties included, have been invited into the studio to discuss. This anticipates disagreement in an institutionally buffered setting (Greatbatch, 1992). The concluding look to the camera is friendly and smiling as if welcoming and implying a safe and encouraging environment for the debate, thus allaying the contradictions verbalized in the talk.

### Summary of the analytic observations

The entrance to the studio space exhibits several purposeful acts to involve the audience in the duties of ongoing action. It addresses the viewers inclusively and invites the audience to *engage in interaction* with the speakers on the screen. This is accomplished by means of gaze, greeting, and visual focalization. The opening talk draws on the *epistemic competence* of the recipients by operationalizing a shared knowledge base which locates participants on a spatio-temporal continuum of events extending the immediate studio space. External events are ongoing and incomplete as the discussion unfolds in the studio. A shared arena of recognition is postulated through assumed knowledge about the news event: the familiarity with the main events and key actors on the scene. The site of ongoing public discussion is approached as an entity that involves participants in a timeline, and prior items in that timeline provide a self-evident basis on which to continue the conversation. Shared cultural knowledge is also employed to enhance mutual familiarity and make sense of the news event in the form of intertextual cultural references (the Wild West, the clash of civilizations etc.).

**Figure 2:**
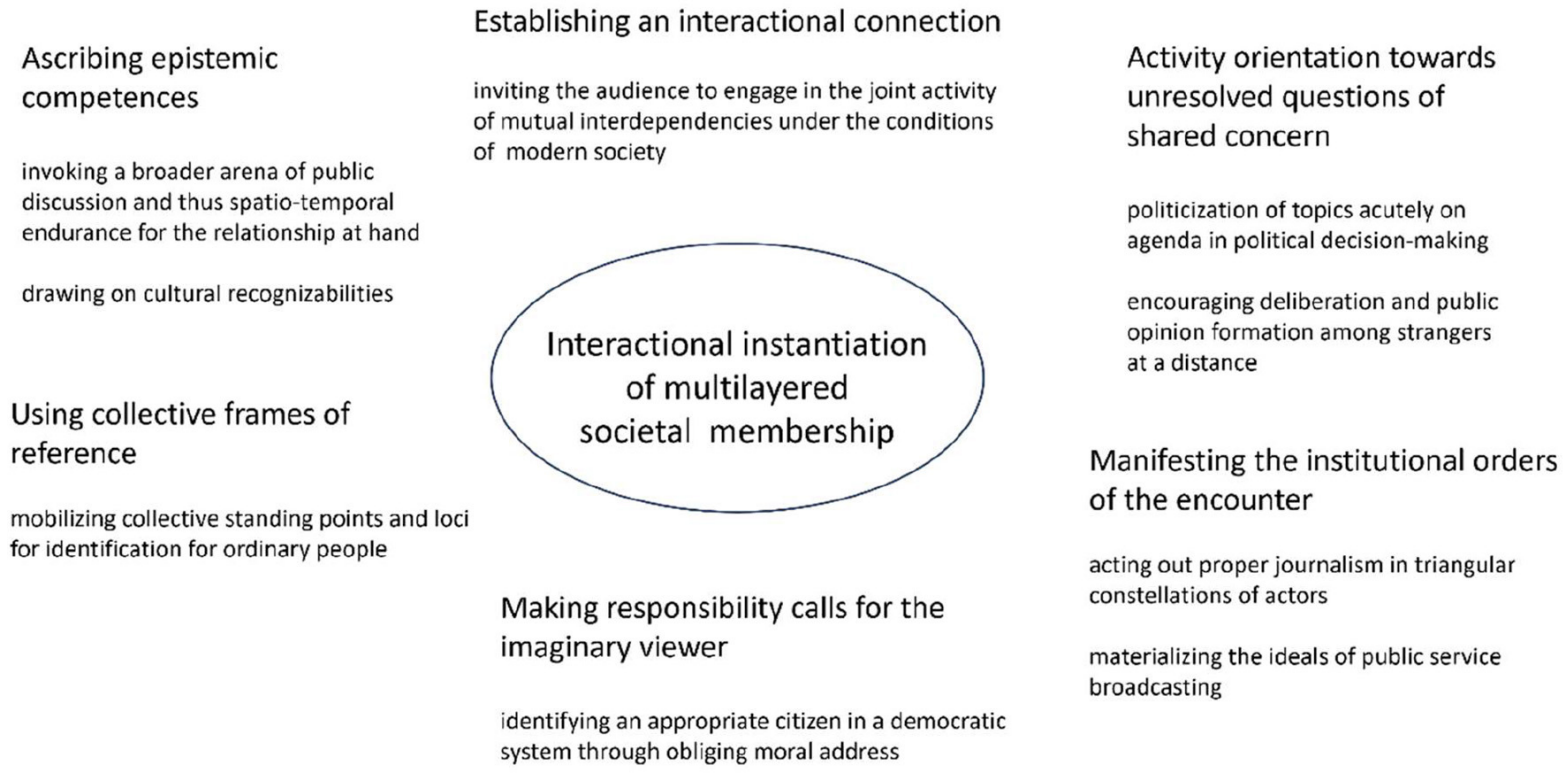
Summary of interactional orientations.

The *activity orientation* in the opening talks aims to address contradictory, uncertain, and unresolved issues in the progress of events. The consequentiality of these decisions is marked as a shared concern, where all those present need to stop and ponder. The audience is addressed as being self-evidently interested and committed to the task. Openness in the face of alternative problematic options instantiates a political scene of action. The act of “politicization” is to open something as political as playable in decision-making (Palonen, 2003). In their opening talk, the journalists raise unresolved questions of shared concern. They organize this task along the lines of institutional appropriateness. Critical alertness, information delivery, and dialogue facilitation are all journalistic virtues through which journalists can perform their institutional tasks. In combination, the acts of inclusive interaction, epistemic positioning, and obliging activity orientation are intended to instantiate and engage an actor category. The invitation to deliberate and take a stance is presented to the viewer through embodied emotional signals and by verbalizing the agency of a generalized collective (such as the country, people, and Finland). These *responsibility calls* invite viewers to become collectively involved in societization and form an opinion on a public matter, invoking the category of modern citizen in the sense-making framework of deliberative democracy (summarized in Figure 2).

## Discussion

Garfinkel (2006) suggested that to find out about the identity of the group, one must explore how the group is “meant” and made meaningful by participants in interaction. This entails investigating the premises of action in the business of coordinating reciprocal recognizabilities through intersubjective activities and orientations. In the spotlight of structuration theory (Giddens, 1984), instances of interaction are always accompanied by structures. The orientations of the journalists in the program anticipate the structural knowledgeability of recipients. These aspects of structuration are evident in the way the opening talk instantiates an extended arena of attention and shared concern, thus enacting a collective and locating the debate in the spatio-temporal continuum of ongoing public discussion on a broader scale. Structural orientations of knowledgeable actors are multilayered. Whereas, conversation analytic interest in the interactional orientations of participants is also interpretative—and for a substantial part based on the cultural competence of the analyst—it is typically targeted at making observations within the boundaries of micro-reality. This article has explored how theoretical conceptualizations can be harnessed to broaden the view, highlight relevancies, and steer analytic attention toward a set of multi-scale recognizabilities on the meso and macro levels of societal activities and orientations as well. The analytic task has nonetheless been to trace the observable markers of sense-making in the concrete details of participants' activities.

Orientations and expectations invoked in the opening address of television discussion draw on the affordances of the technologically mass-mediated broadcast arena. The formation of the participation framework, the management and coordination of participant roles is a combination of technologically facilitated communicative reach, technical means, topics, and forms of address (Hutchby, 2014). The analysis above identified several interactional features that turn the opening talk into an inclusive, audience-involving invitation to get along and “act on society”. The societal membership enacted in the opening infuses several multi-scale structural frames of action. It is not just a channel that reaches far but an arena which invites countless groups of unknown strangers into the interaction, calling for a response and anticipating agency in the system. The talk covers a news item, yet rather than delivering knowledge, it is about allocating epistemic competence in despatialized simultaneity, anticipating a shared base and the endurance of the relationship. Rather than reporting about the openness of events, the talk encourages the recipient to consider the options and take responsibility in deliberating solutions to matters of shared concern.

Nested frames of modern co-existence, democratic political system and public service media format are structured by means of relevant activity orientations, epistemic positionings, and collective responsibility calls in the broadcast opening talk. Thus, alongside addressing broader commitments to mutual interconnections and legitimating work through public deliberation, the opening manifests institutional relevancies structuring the immediate site of interaction. The journalists moderating the debate make sure to convey that their response to the topic of talk adheres to the normative expectations embedded in their institutional role. In the piece of opening talk, journalists play the key role, but they do not stand alone. The role enacted by the journalist is interrelational and connected to other identifications in the program (Rautajoki, 2009). The way journalists position themselves in interaction is by projecting positionings in despatialized simultaneity to other parties of the encounter. Thus, the tasks and responsibilities of the audience are projected in the reciprocal dynamic of opening identifications. To follow Garfinkel, identities acquire meaning in specific locations of action through orderly produced recognizability, in which “by your actions you tell me who I am, and by my actions I tell your who you are” (Rawls, 2006, p. 77).

The findings of this research do not say that acts of societization happen exclusively, primarily, or specifically in the arena of television discussions or by deploying these interactional means only. Instead, the findings demonstrate how the realization of societal membership in modern mass-scale society recurs unnoticeably in the routines of everyday life and how this task is managed in the activity context of discussion programs. Society is not only a location or a world of meaning. The concept of societization brings to light that society is also about activities: reproduction and maintenance by doing. The members of society make it real in the routinized practices of everyday life, willingly and skillfully. These situated acts of accomplishing society are so routinized that they easily go unnoticed. In the punctuated practices of daily life, one may sit down on the sofa and turn on a current affairs TV program, expecting to hear news *about* society and carrying on these activities *within* society, while paying no attention to the fact that in these fleeting moments, one actually takes part in instantiating and reproducing the thing called society. The set of orientations and activities observed in the opening talk cultivates a sense of collective and includes anonymous individual recipients, which serves integrative functions. The combination of theoretical perspectives and micro-analytic tools has been useful in explicating the crucial function of interactional methods in exercising mass-scale societization in broadcast talk.

Broadcast research has underlined the potential of broadcast talk to build communicative relationships (Scannell, 1989). Who is saying what to whom about whom is often as interesting in media discourse as what is said (Fairclough, 1995). Viewing identifications from the broader angle of distributed participation prompts the question about their extended implications. Detailed analysis of the relationships mobilized in media talk opens up a view into the manifestation of power (Hutchby, 2006). It is a scenery that takes us back to wonder what exactly is accomplished by the relational constellation of the opening talk. From the perspective of relational power (Foucault, 1980), the multilayered relevancies participants orient to in the program openings intersect with power practices. It is to approach power processually from a “transactional perspective” (Selg, 2018), according to which power appears in interaction in the form of functional effects traveling through the acts of participation in the collaborative efforts to bring about intelligible actions. In this view, situated action transports power relations manifested in identifications. Actor identities are constituted in the interplay of situated and enduring elements, drawing on structural recognizabilities, actualizing in cooperation and resulting in a set of social orders. At the interface of action and structure, a “cultural apparatus of effective relationships” to make sense of and move in social situations (Deleuze, 1992) is not that far from the principle of approaching “cultural categorizations as an inference-making machine” (Sacks, 1995). This cultural set-up is not tantamount to pre-determination but leaves room to maneuver. The associative fabric of inferences and normative expectations connected to membership categorizations is what makes them so resourceful in obliging address and persuasion. It makes it possible to scaffold claims and make normative calls and argumentative moves on relational premises (Rautajoki, 2022).

Talking modern society *into being* is interrelational and rooted in particular collaborative materializations of normative cartographies at specific points in time. The focus of this article has been on the arena of television discussions. It has been proposed in earlier research to call journalists “practitioners of society” to facilitate interaction between subgroups in society (Pietilä, 2011). Journalism is about practicing societization, no doubt; yet, the wording seems ignorant of the relational set-up journalists convey while channeling social co-existence and political opinion formation. The arena of television discussions instigates public deliberation, while it also stands to strengthen the status and prominence of public service journalism at the center of modern mass-scale democracy. In good and in bad, it facilitates societization in and through a specific relational format, advancing political agency and integration yet channeling these activities in the landscape and on the terms of a given institutional entity. All in all, rather than talk about a framework, the dynamic of participation in this arena might be more aptly described as a *participation field* activated for the participants to take action in. This field of action is actualized through the interactional orchestration of multilayered structural orientations, instantiating multiple memberships and respective category-bound responses. Participants are invited to navigate multi-scale normative frameworks and positionings in their acts of societization: to engage in quasi-interactional connection, get involved in the activity of public opinion formation and act out the actor categories of a public service journalist and an alert citizen. For participants in social interaction, this comes naturally. Micro-analytic tools have been valuable in highlighting the detailed means through which this complexity is managed.

To conclude, why does it matter to see society as an intentional achievement rather than a pre-existing spatial frame for action? Because it points to the ultimate contingency and also to the vulnerability of societal membership. Practices that have become self-evident should not be taken for granted. The “functioning we” can be populated with various imageries, intentions, and principles (Rautajoki and Fitzgerald, 2022). Unfortunately, impersonalized intersubjective intent to *reach across differences* is not the only possible way to organize interchange among collectives. More and more public address is “opting out” of these principles, for example, in the antagonism of right-wing populist rhetoric. It is worth contemplating what is at stake when compromising the premises of modern pluralistic society. Analyzing the accomplishment of mass scale “we” enhances understanding of who “we” involves in talk and how it emerges through various forms of address. It foregrounds the question of how severely the current trends in public discourse are undermining the orders of societal membership. And what is to follow from their success? One is left wondering how detrimentally one-eyed antagonism is now talking a form of collective co-existence *out of being*.

## Data availability statement

The original contributions presented in the study are included in the article/supplementary material. Further inquiries can be directed to the corresponding author.

## Ethics statement

Ethical approval was not required for the study involving human data in accordance with the local legislation and institutional requirements. Written informed consent was not required for participation in the study or for the publication of directly/indirectly identifiable data.

## Author contributions

The author confirms being the sole contributor of this work and has approved it for publication.
